# Gastrojejunocolic fistula after gastrojejunostomy: a case series

**DOI:** 10.1186/1752-1947-2-193

**Published:** 2008-06-04

**Authors:** Jin-Ming Wu, Ming-Yang Wang, Po-Huang Lee, Ming-Tsan Lin

**Affiliations:** 1 National Taiwan University Hospital, Department of Surgery, Taipei, Taiwan

## Abstract

**Introduction:**

Gastrojejunocolic (GJC) fistulae represent a significant post-surgical cause of morbidity and mortality. GJC fistulae represent rare post-surgical complications, and most are associated with gastric surgery. In the past, this complication has been under-recognized because a fistula may form years after surgery.

**Case presentation:**

We describe two cases of gastrojejunocolic fistula in men aged 67 and 60 who both initially presented with watery diarrhea and weight loss. Upper GI studies with small bowel follow-through or barium contrast enema studies allowed a conclusive diagnosis to be made. Both patients underwent one-stage en bloc resection, and their postoperative course was uneventful.

**Conclusion:**

With surgery, this condition is entirely correctable. Pre-operative nutritional status should be evaluated in patients undergoing corrective surgery, and total parenteral nutrition plays a major role in the provision of bowel rest to allow recovery in malnourished patients.

## Introduction

Gastrojejunocolic (GJC) fistulae represent a significant post-surgical cause of morbidity and mortality. In the past, this complication has been under-recognized because a fistula may form years after surgery. We describe two cases of GJC fistula in patients who both underwent a single-stage correction, and we review the literature relevant to their diagnosis and management.

## Case presentation

### Case 1

A 67-year-old man presented with gastric perforation secondary to an eroding gastric ulcer. He underwent a primary repair in 1963. His post-surgical course had previously been complicated by pyloric stenosis after a gastrojejunostomy and truncal vagotomy in 1998. He presented with a 2-month history of approximately 10 episodes per day of watery diarrhea that occurred immediately after meals and he had experienced weight loss of 8 kg during that time. Hemoglobin was slightly low at 12.3 g/dl (normal range, 13 to 15 g/dl); albumin was slightly low at 2.8 g/dl (normal range, 3.5 to 5.5 g/dl); total protein was normal. Both fecal leukocyte and occult blood tests were negative. Stool cultures for *Shigella*, *Salmonella *and viral pathogens were all negative. Colonoscopy was remarkable for colitis at the distal transverse colon, but no fistula was noted.

**Figure 1:**
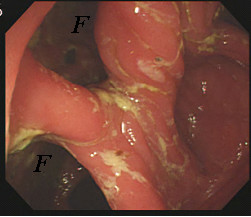
Colonoscopic findings reveal two fistulae (*F*) at the distal transverse colon.

**Figure 2:**
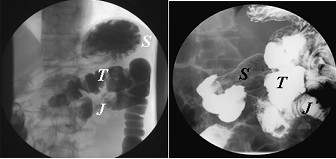
**Both the urograffin enema and barium meal confirmed the diagnosis of gastrojejunocolic fistula.** Left: The Urograffin enema demonstrating early contrast filling of the stomach and jejunum.*S*, stomach; *J*, jejunum; *T*, transverse colon. Right: The barium meal shows the jejunum and colon simultaneously.

**Figure 3:**
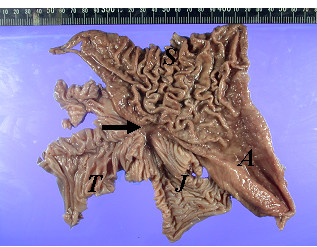
**Macroscopic view of the resected specimen.** The fistula measures 1 cm in diameter. *S*, stomach; *A*, antrum; *J*, jejunum;*T*, transverse colon.

Biopsy was performed at the site of active inflammation and this unexpectedly demonstrated small bowel mucosa. During a subsequent colonoscopy, at least two fistulae were identified in the transverse colon (Figure 1). Both gastrograffin enema (Figure 2, left) and upper gastrointestinal (GI) series with small bowel follow-through (Figure 2, right) delineated the extent of the GJC fistulae. Bowel rest with nutritional support via total parenteral nutrition (TPN) was administered, and elective surgical correction was performed. Intra-operative findings indicated severe adhesion between the greater curvature of the stomach, proximal jejunum, and transverse colon. As a result, the patient underwent one-stage en bloc resection: subtotal gastrectomy and segmental resection of the jejunum with a Roux-en-Y anastomosis and segmental resection of the transverse colon with side-to-side anastomosis. Histological examination found no evidence of active ulcers or malignant transformation within the fistulae (Figure 3).

### Case 2

A 60-year-old man presented with a 3-week history of diarrhea and weight loss. He had undergone subtotal gastrectomy with Billroth-II reconstruction 2 years previously because of peptic ulcer disease. Hemoglobin was slightly low at 11.9 g/dl (normal range, 13 to 15 g/dl); albumin was slightly low at 2.8 g/dl (normal range, 3.5 to 5.5 g/dl); total protein was normal. Gastroscopy found an anastomotic ulcer. Colonoscopy revealed edematous change of the colonic mucosa at the splenic flexure, but no fistular orifice was noted. An upper GI series with small bowel follow-through demonstrated the presence of the anastomotic ulcer as well as a fistula between the afferent jejunum and transverse colon. He underwent revision gastrectomy and segmental resection of the jejunum and transverse colon with Roux-en-Y reconstruction. The histological findings revealed that the fistula, which measured 7 cm, occurred adjacent to an active ulcer. Recovery was uneventful and the patient remained well at follow-up.

## Discussion

GJC fistula is an uncommon complication after gastrojejunostomy. GJC fistulae may occur postoperatively in the context of either peptic ulcer or malignant GI disease. In the past, GJC fistulae have often involved serious complications and have been associated with high mortality because of the poor nutritional status of affected patients [1]. Staged repair of GJC fistulae was initially favored to decrease mortality [2-4]. After the introduction of parenteral nutrition and intensive care in the 1970s, more patients with GJC fistulae were able to undergo elective one-stage en bloc resection as originally advocated by Marshall and Knud-Hansen [5]. Most patients could tolerate the operation well without the need for postoperative care.

The diagnosis of a GJC fistula is typically straightforward if clinical suspicion is high. Marshall and Knud-Hansen [5] described the triad of symptoms associated with a GJC fistula as diarrhea, weight loss, and eructation of fecal-smelling gas. No eructation of fecal-smelling gas was noted in our cases, but immediate diarrhea after oral intake may suggest gastrocolonic fistulae. Some patients reported undigested food in the stools if the size of a GJC fistula was large.

If GJC fistulae are suspected, an upper GI series or water-soluble contrast enema may confirm the diagnosis. Barium enema has been found by Thoeny et al. [6] to have a 95% sensitivity for making the diagnosis compared with a 27% sensitivity with X-ray film series of the upper GI tract. In both of our cases, upper GI series confirmed the diagnosis. The nature of the fistula tract varied, and a computed tomography scan may supplement both this information (and demonstrate pathology such as an abscess, cancer or ulcer) and that of the anatomy adjacent to the fistula.

Endoscopy may also be a helpful tool in establishing the diagnosis, and can exclude other GI disease. Nussinson et al. [7] previously found that simultaneous examination using gastroscopy and colonoscopy was useful in the diagnosis of GJC fistulae. In our cases, neither gastroscopy nor colonoscopy was able to detect the fistulae initially, probably because of incomplete preprocedural bowel preparation. A second colonoscopy in the first case demonstrated the fistulae under a clear examination field and with serial air insufflation. These findings highlight the fact that endoscopy is an operator-dependent diagnostic tool, and negative findings are insufficient to rule out the diagnosis of GJC fistulae. However, in one of our cases, tissue biopsy provided clues about the presence of fistulae once small intestinal mucosa were detected histologically.

GJC fistula is thought to be a late complication of inadequate surgery, resulting from gastroenterostomy, inadequate gastric resection, or incomplete vagotomy. Ulcers are believed to contribute to the formation of a GJC fistula. If a stomach ulcer occurred, it may contribute to early formation of a GJC fistula. This could explain why the duration varied in our cases. With the use of eradication therapy for *Helicobacter pylori*, the incidence of GJC fistulae may be expected to decrease. However, other contributory factors exist that may be increasing including the rising proportion of elderly or malnourished patients, or patients with cancer, potentially leading to postoperative complications. As a result, GJC fistula should be kept in the differential diagnosis if diarrhea persists in post-gastric-bypass patients immediately after oral intake.

## Conclusion

GJC fistulae have historically been considered as rare complications after gastric surgery. They may take considerable time to develop, and have been observed more than 20 years after the relevant operation. Therefore, the potential contribution of previous surgery is often overlooked. Patients with a GJC fistula often present with watery diarrhea immediately after oral intake, as well as malnutrition. Diagnosis is straightforward if GJC fistula is suspected. Upper GI series with small bowel follow-through or water-soluble contrast enema study appear to be more sensitive diagnostic tools than endoscopy. Negative findings on endoscopy do not rule out the diagnosis of a GJC fistula. One-stage en bloc resection is feasible if the patient's general condition is good or can be maintained during a time of bowel rest with TPN.

## Abbreviations

GI: gastrointestinal; GJC: gastrojejunocolic; TPN: total parenteral nutrition.

## Competing interests

The authors declare that they have no competing interests.

## Consent

Written informed consent was obtained from the patients for publication of these case reports and any accompanying images. A copy of the written consent is available for review by the Editor-in-Chief of this journal.

## Authors' contributions

All authors contributed to each stage of this work. JMW, MYW and PHL contributed equally to the work.
